# Diversity of glycosil hydrolase family 18 genes in environment isolates of the entomopathogenic fungus *Metarhizium anisopliae*

**DOI:** 10.1186/1753-6561-8-S4-P111

**Published:** 2014-10-01

**Authors:** Nicolau Sbaraini, Ângela Junges, Augusto Schrank

**Affiliations:** 1 Centro de Biotecnologia da Universidade Federal do Rio Grande do Sul (CBiot/UFRGS), Programa de Pós-graduação em Biologia Celular e Molecular (PPGBCM), Av. Bento Gonçalves, 9500, Bairro Agronomia, CEP 91501-970, Porto Alegre, Rio Grande do Sul, Brazil

## Backgound

*Metarhizium anisopliae *is a model for host-pathogen studies due to its ability to infect several different arthropods. The first barrier to accomplish successful host-infection is transverse the host cuticle, which is a rigid chitin-rich structure. To surpass this barrier, the fungus produces several hydrolytic enzymes, among which are chitinases and endo-β-N-acetylglucosaminidases, glycosil hydrolase 18 (GH18) members [1,2]. In fungi, GH18 enzymes have nutritional importance and exhibit morphogenic and autolytic functions, acting at different processes of fungal development and life cycle maintenance. Assigning functional role for these genes in each process is one of the goals in entomopathogenic fungi study [3]. A genomic analysis performed in our laboratory, in *M. anisopliae *E6 strain, identified twenty-three GH18 putative genes [3-5]. Considering this variety, this study aims to evaluate the diversity of these genes amongst *M. anisopliae *strains, to access their distribution in environmental isolates of the fungus. DNA samples from 23 *M. anisopliae *strains (CG291, NORDESTE, CARO7, CG125, CG343, CG374, CG46, CARO12, CARO14, CARO19, CG30, CG97, CG320, AL, MT, M5, CARO11, CARO15, CARO16, CG47, PL57, CG87 e CG491) isolates from different arthropods and places were subjected to PCR analysis to evaluate the presence of each of the 23 GH18 putative genes found in the genome of strain E6.

## Methods

All strains were grown on Cove's Complete Medium (MCc) agar plates at 28°C until sporulation. Spores were harvested with 0.01% Tween solution and inoculated into liquid MCc cultured in a rotatory shaker at 28ºC, 180 rpm for 48 hours. After growth, DNA was extracted using lysis solution and phenol/chloroform method. The presence of GH18 genes was detected by PCR, using primers designed for *M. anisopliae *strain E6 genes.

## Results and conclusions

From the analysis it is possible to suggest that these GH18 genes sequences are well conserved in other strains. We identified 17 *M. anisopliae *strains with possible absence of one or more GH18 genes. Amongst the most prominent are strains lacking five genes. *ChimaA1, chimaA2, chimaA4, chimaA8, chimaB4, chimaB6, chimaD1 *and *chimaD2 *putative genes were detected in all strains studied. Furthermore, the *chimaA7 *gene was not detected in eight strains and the *chimaB5 *gene was also not detected in six strains. Strains with the initials "CARO" from Mexico displayed higher number of absences, compared to E6 isolated in Brazil. Geographical distance is one of the possible factors that could contribute for the divergence in these strains. Although the absence of genes in some strains does not necessarily imply ortholog absence, because the primers used in this work were constructed based on E6 strain sequences, our results suggest diversity of GH18 members amongst *M. anisopliae *isolates. The possible absence of GH18 genes may result in differences in development, morphology and pathogenicity in the fungus that are now under study.

## References

[B1] A Vainstein M H Metarhizium anisopliae enzymes and toxins Toxicon 2010 56 7 1267 74 10.1016/j.toxicon.2010.03.008 20298710

[B2] GD Tzelepis Melin P Jensen DF Stenlid J Karlsson M Functional analysis of glycoside hydrolase family 18 and 20 genes in Neurospora crassa Fungal Genet Biol 2012 49 9 717 30 10.1016/j.fgb.2012.06.013 22796096

[B3] Junges A Metarhizium anisopliae: Expressão de proteína toxica de origem vegetal e analise genômica de quitinases Programa de Pós-Graduação em Biologia Celular e Molecular, Universidade Federal do Rio Grande do Sul 2010

[B4] Karlsson M Stenlid J Comparative evolutionary histories of the fungal chitinase gene family reveal non-random size expansions and contractions due to adaptive natural selection Evol Bioinform Online 2008 4 47 60 19204807 10.4137/ebo.s604 PMC2614207

[B5] V Huemer B Seiboth B Kubicek C P A complete survey of Trichoderma chitinases reveals three distinct subgroups of family 18 chitinases FEBS J 2005 272 22 5923 39 10.1111/j.1742-4658.2005.04994.x 16279955

